# Decreased miR122 in hepatocellular carcinoma leads to chemoresistance with increased arginine

**DOI:** 10.18632/oncotarget.3234

**Published:** 2015-03-21

**Authors:** Takahiro Kishikawa, Motoyuki Otsuka, Poh Seng Tan, Motoko Ohno, Xiaochen Sun, Takeshi Yoshikawa, Chikako Shibata, Akemi Takata, Kentaro Kojima, Kenji Takehana, Maki Ohishi, Sana Ota, Tomoyuki Noyama, Yuji Kondo, Masaya Sato, Tomoyoshi Soga, Yujin Hoshida, Kazuhiko Koike

**Affiliations:** ^1^ Department of Gastroenterology, Graduate School of Medicine, The University of Tokyo, Tokyo 113–8655, Japan; ^2^ Japan Science and Technology Agency, PRESTO, Kawaguchi, Saitama 332–0012, Japan; ^3^ Liver Cancer Program, Tisch Cancer Institute, Division of Liver Diseases, Department of Medicine, Icahn School of Medicine at Mount Sinai, NY 10029, USA; ^4^ Division of Gastroenterology and Hepatology, University Medicine Cluster, National University Health System, 119228, Singapore; ^5^ Pharmacology Research Laboratory, Research Institute, Ajinomoto Pharmaceutical Co., Ltd., Kawasaki, Kanagawa 210–8681, Japan; ^6^ Institute for Advanced Biosciences, Keio University, Tsuruoka, Yamagata 997–0052, Japan

**Keywords:** HCC, miR122, SLC7A1, nitric oxide, arginine

## Abstract

Reduced expression of microRNA122 (miR122), a liver-specific microRNA, is frequent in hepatocellular carcinoma (HCC). However, its biological significances remain poorly understood. Because deregulated amino acid levels in cancers can affect their biological behavior, we determined the amino acid levels in miR122-silenced mouse liver tissues, in which intracellular arginine levels were significantly increased. The increased intracellular arginine levels were through upregulation of the solute carrier family 7 (SLC7A1), a transporter of arginine and a direct target of miR122. Arginine is the substrate for nitric oxide (NO) synthetase, and intracellular NO levels were increased in miR122-silenced HCC cells, with increased resistance to sorafenib, a multikinase inhibitor. Conversely, maintenance of the miR122-silenced HCC cells in arginine-depleted culture media, as well as overexpression of miR122 in miR122-low-expressing HCC cells, reversed these effects and rendered the cells more sensitive to sorafenib. Using a reporter knock-in construct, chemical compounds were screened, and Wee1 kinase inhibitor was identified as upregulators of miR122 transcription, which increased the sensitivity of the cells to sorafenib. These results provide an insight into sorafenib resistance in miR122-low HCC, and suggest that arginine depletion or a combination of sorafenib with the identified compound may provide promising approaches to managing this HCC subset.

## INTRODUCTION

HCC is the third most common cause of cancer-related mortality worldwide [1]. Although advances in early detection and novel therapies have improved prognosis, no effective therapy other than the multi-kinase inhibitor, sorafenib [2], is currently available for advanced disease [3].

The expression of miR122, a liver-specific microRNA (miRNA), is frequently repressed in human HCC [4], and this is functionally linked with aggressive phenotypes in HCC cells [5, 6]. Mice lacking the gene encoding miR122 suffer from liver steatosis and HCC, suggesting a critical role of miR122 in metabolic homeostasis and oncogenesis in the liver [7, 8]. *In vivo* studies inhibiting miR122 function also showed effects on fatty acid and iron metabolism [7, 9–13], suggesting that miR122 had pleiotropic metabolic effects [14].

The principal function of miRNAs is the posttranscriptional regulation of gene expression by base pairing to their target mRNAs. Although numerous genes have been recognized as candidates, few have been confirmed experimentally as direct targets of miR122. Among the genes identified, cationic amino acid transporter member 1 (CAT1, also known as solute carrier family 7, SLC7A1) is the best-known direct target of miR122. Because SLC7A1 is a well-known arginine transporter [15], it was hypothesized that repressed miR122 expression in HCC would lead to deregulated levels of intracellular amino acids, especially arginine, which may affect the biological phenotype of HCC.

Sorafenib is currently the only systemic treatment for patients with advanced HCC. However, the survival advantage is only 2.8 months [2]. Therefore, enhancing its efficacy or devising effective combination therapies are urgently needed [16]. In this study, changes in amino acid levels in miR122-silenced mouse liver tissues, caused by impaired miR122 function, were first assessed. Next, based on the results, chemoresistance of HCC cells with impaired miR122 function against sorafenib was determined. Finally, a comprehensive screen was performed of chemical compounds that increased miR122 expression levels in HCC and that could alleviate the observed resistance to sorafenib. From these results, we proposed possible interventional methods for a subset of HCCs with repressed miR122 levels.

## RESULTS

### Intracellular arginine and NO levels were increased in miR122-silenced HCC cells

Because SLC7A1 is a direct target of miR122 *in vitro* [17] and miR122 expression levels are frequently decreased in HCC tissues [6, 18, 19], we first analyzed the genome-wide mRNA and miRNA profiles of clinical HCC tumors infected with chronic hepatitis B (*n* = 192) and hepatitis C (*n* = 89) using the public data [20–23] to determine the correlation in the expression levels of between miR122 and SLC7A1 in clinical HCC samples. Expression levels of miR122 and SLC7A1 were negatively correlated, modestly but significantly, in both human cohorts ( *p* < 0.001) (Supplementary Figure 1a and 1b).

**Figure 1:**
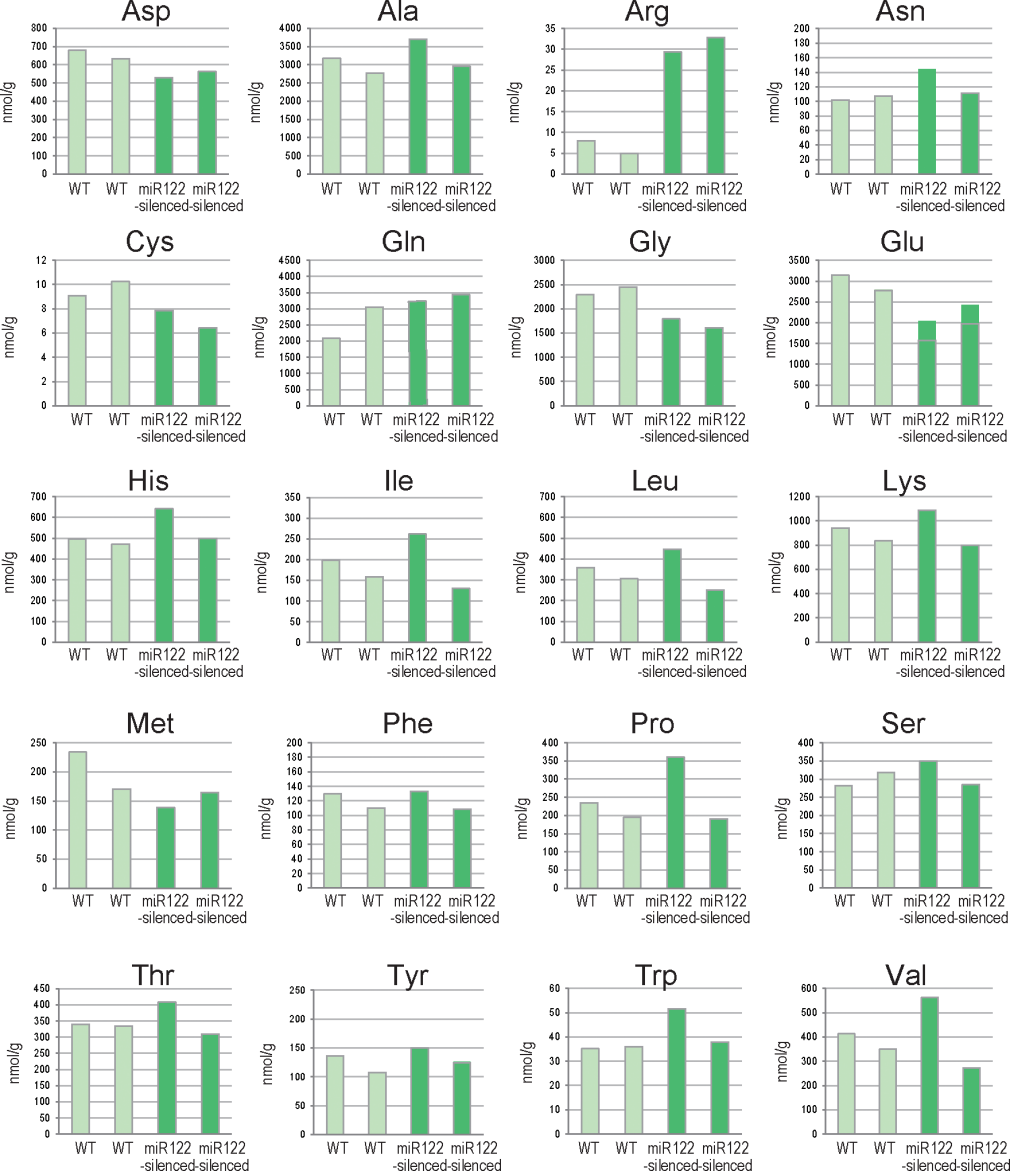
Changes in amino acid levels in liver tissues from miR122-silenced mice Results of amino acid analyses of liver tissues from control and miR122-silenced transgenic mice (*n* = 2 each). WT, control mice; miR122-silenced, miR122-silenced transgenic mice.

Recently, metabolomic profiling has revealed a number of perturbed metabolic pathways, including amino acids, in cancers [24, 25]. To determine the biological effects of impaired miR122 function on amino acid levels, the latter were comprehensively quantified in miR122-silenced mouse liver tissues [5]. Of the 20 amino acids examined, arginine showed reproducible differences in levels in between the control and miR122-silenced liver tissues from two individuals per group (Figure 1). Because arginine is an amino acid which is transported into cells via SLC7A1 [26], a target of miR122, we focused on the reproducibly increased arginine content of miR122-silenced tissues, in subsequent studies.

**Figure 2:**
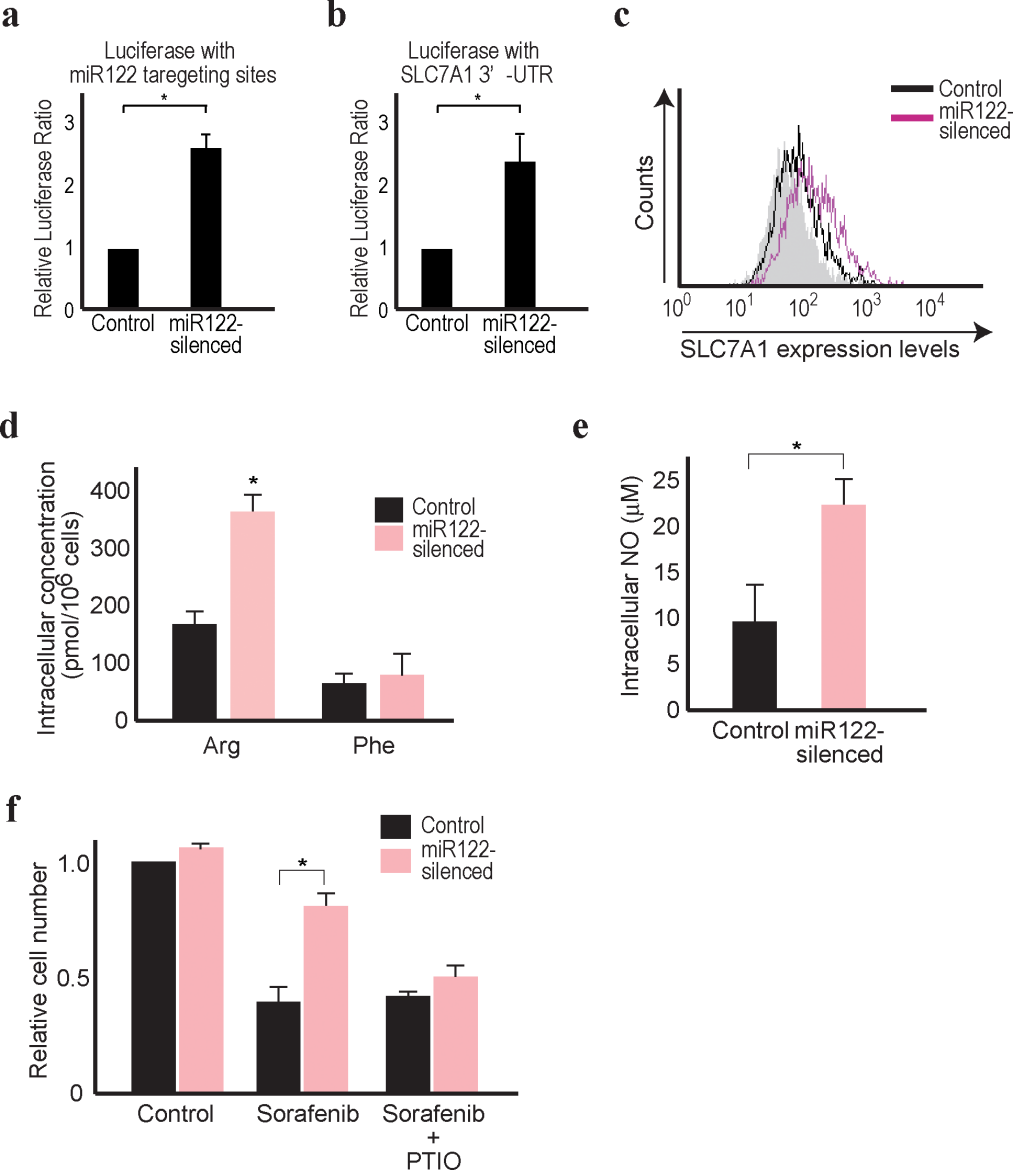
Reduced miR122 is linked with resistance against sorafenib **(a)** The firefly luciferase-based reporter with miR122 3′UTR responsive elements was transfected along with the Renilla luciferase-based control reporter. Firefly luciferase values were normalized to those of Renilla luciferase (as an internal control) to calculate the relative luciferase values. Control cell values were set to 1. Data represent the means ± s.d. of three independent experiments. *, *p* < 0.05. **(b)** The Renilla luciferase-based reporter with the SLC7A1 3′UTR (pRL-catA) was transfected as well as the CMV-driven firefly luciferase-based control reporter, to determine the effect of miR122 on the SLC7A1 3′UTR. Renilla luciferase values were normalized to those of firefly luciferase (as an internal control) to calculate the relative luciferase values. Control cell values were set to 1. Data represent the means ± s.d. of three independent experiments. *, *p* < 0.05. **(c)** SLC7A1 expression was increased in miR122-silenced Huh7 cells. Flow cytometry assessment of SLC7A1 protein expression in control cells (black line) and miR122-silenced cells (pink line) was performed. Gray-shaded histograms represent the isotype IgG background staining. Representative results from three independent experiments are shown. **(d)** Intracellular levels of arginine, but not phenylalanine, were increased in miR122-silenced cells. Data represent the means ± s.d. of three independent experiments. *, *p* < 0.05. **(e)** Intracellular NO levels were increased in miR122-silenced cells. Data represent the means ± s.d. of three independent experiments. *, *p* < 0.05. **(f)** The effects of sorafenib were inversely correlated with the expression levels of miR122. Relative cell numbers were counted after treatment for 48 h with 5 μM sorafenib with or without PTIO in miR122-silenced Huh7 cells. The value of control cells with DMSO treatment was set to 1 *, *p* < 0.05 (*n* = 3).

To confirm the above screening results *in vitro*, we used miR122-silenced Huh7 cells, well-differentiated HCC cells which stably express miR122 antisense constructs and have impaired miR122 function [5] (Figure 2a). Huh7 cells were chosen for the experiments using the antisense miR122 construct because they have endogenously high miR122 expression levels [27] and the effects of miR122-silecing are more easily observed. Consistent with previous reports [27], luciferase expression from a reporter construct containing the 3′ UTR of the SLC7A1 gene was increased, and SLC7A1 expression was increased by miR122-silecing (Figure 2b and 2c). Moreover, while intracellular levels of phenylalanine did not change, intracellular arginine levels increased significantly, confirming the initial screening results (Figure 2d).

Arginine is an endogenous substrate for the production of intracellular NO [28]. Intracellular NO levels were elevated in miR122-silenced Huh7 cells without deregulated NO synthetase expression (Figure 2e and Supplementary Figure 2). Because NO has pleiotropic effects in tumor cells such as chemotherapeutic resistance and evasion of apoptosis [29–31], we focused on the chemoresistance against sorafenib, the only systemic treatment for HCC currently approved. As expected, miR122-silenced cells showed more resistance to sorafenib, determined by the number of the survival cells after sorafenib treatment for 48 hrs (Figure 2e). These effects appeared NO dependent, because the resistance was canceled by the treatment of carboxy-PTIO, a potent NO remover (Figure 2f). Because increased intracellular NO synthesis is also a property of cancer-progenitor cell-like features [30, 32–34], the expression levels of HCC cancer-progenitor cell markers, such as EpCAM and CD13 [35] were examined, which were significantly increased in miR122-silenced cells (Supplementary Figure 3). These results suggested that miR122-silenced HCC cells were more resistant to sorafenib because they contained higher intracellular arginine and NO levels, possibly through elevated expression of SLC7A1, an arginine transporter and a target of miR122.

### Arginine depletion blocked upregulation of intracellular NO levels in miR122-silenced HCC cells

**Figure 3:**
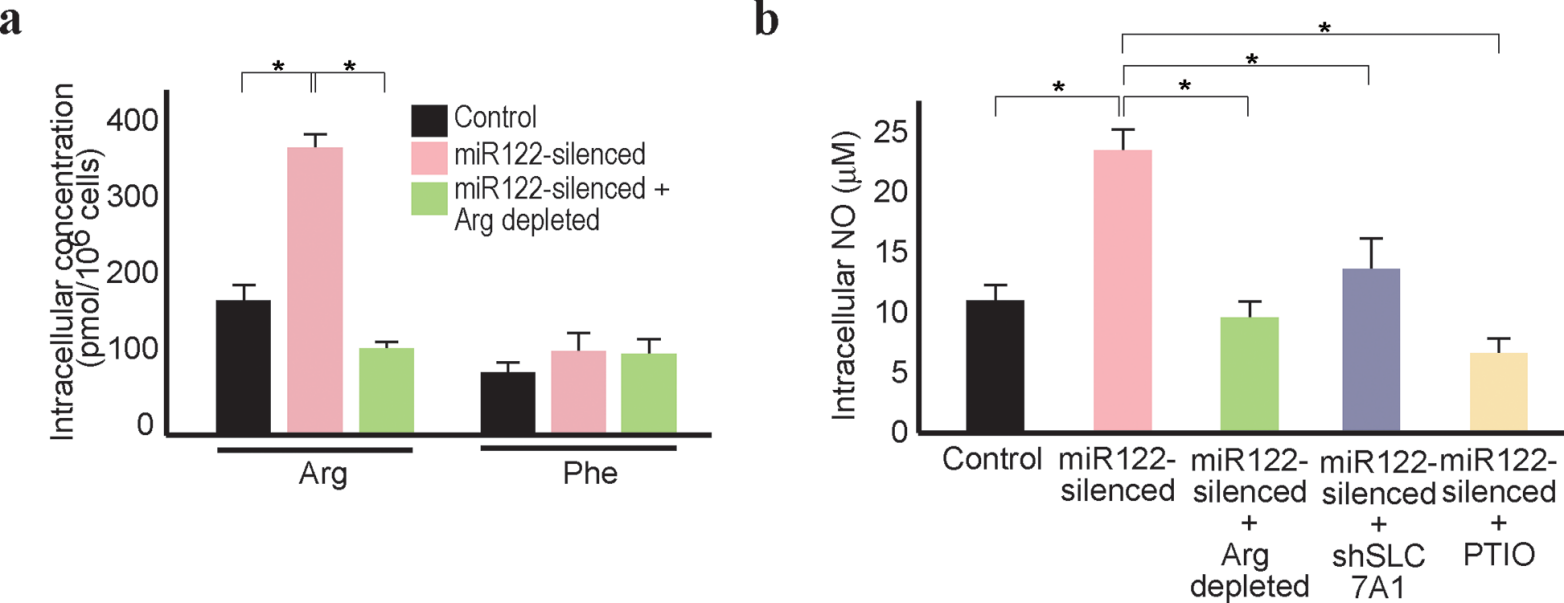
Arginine depletion decreases NO levels in miR122-silenced Huh7 cells **(a)** Intracellular arginine levels decreased in miR122-silenced cells under arginine-depleted conditions. *, *p* < 0.05 (*n* = 3). **(b)** Intracellular NO levels decreased in miR122-silenced cells under arginine-depleted conditions. This effect was reversed by knockdown of SLC7A1. Control cells (control), miR122-silecend cells (miR122-silenced), miR122-silenced cells cultured in arginine-depleted media (miR122-silenced + Arg depleted), and miR122-silenced cells with expression of shSLC7A1 (miR122-silenced + shSLC7A1) were tested. Cells treated with PTIO to remove NO were included as a positive control. *, *p* < 0.05 (*n* = 3).

Because intracellular arginine levels are largely dependent on the transport of extracellular arginine into cells [36], the effects of arginine depletion in the culture media were examined. Although intracellular arginine levels were increased in miR122-silenced cells in the arginine-content media, this increase was not seen when the cells were cultured under arginine-depleted conditions (Figure 3a). Accordingly, culture under arginine-depleted conditions prevented an increase in intracellular NO, with levels similar to those of the control cells treated with a NO remover (Figure 3b). In addition, because knockdown of SLC7A1 in miR122-silenced cells through expression of shSLC7A1 also prevented an increase in intracellular NO, it was considered that the increased NO levels in miR122-silenced cells were due to increased SLC7A1 levels (Figure 3b). Concordantly, although we could not test the effects of sorafenib under arginine-depleted media because most cells died when maintained in serum-free media (to remove the effects of arginine in serum) and treated for 48 h with sorafenib, the expression levels of HCC cancer-progenitor cell-markers were decreased by arginine depletion (Supplementary Figure 3b). These results showed that reduced intracellular arginine and NO levels in miR122-silecend cells can be achieved by the depletion of arginine in the extracellular media.

### Forced miR122 expression in HCC cells decreased SLC7A1 expression and intracellular NO levels

**Figure 4:**
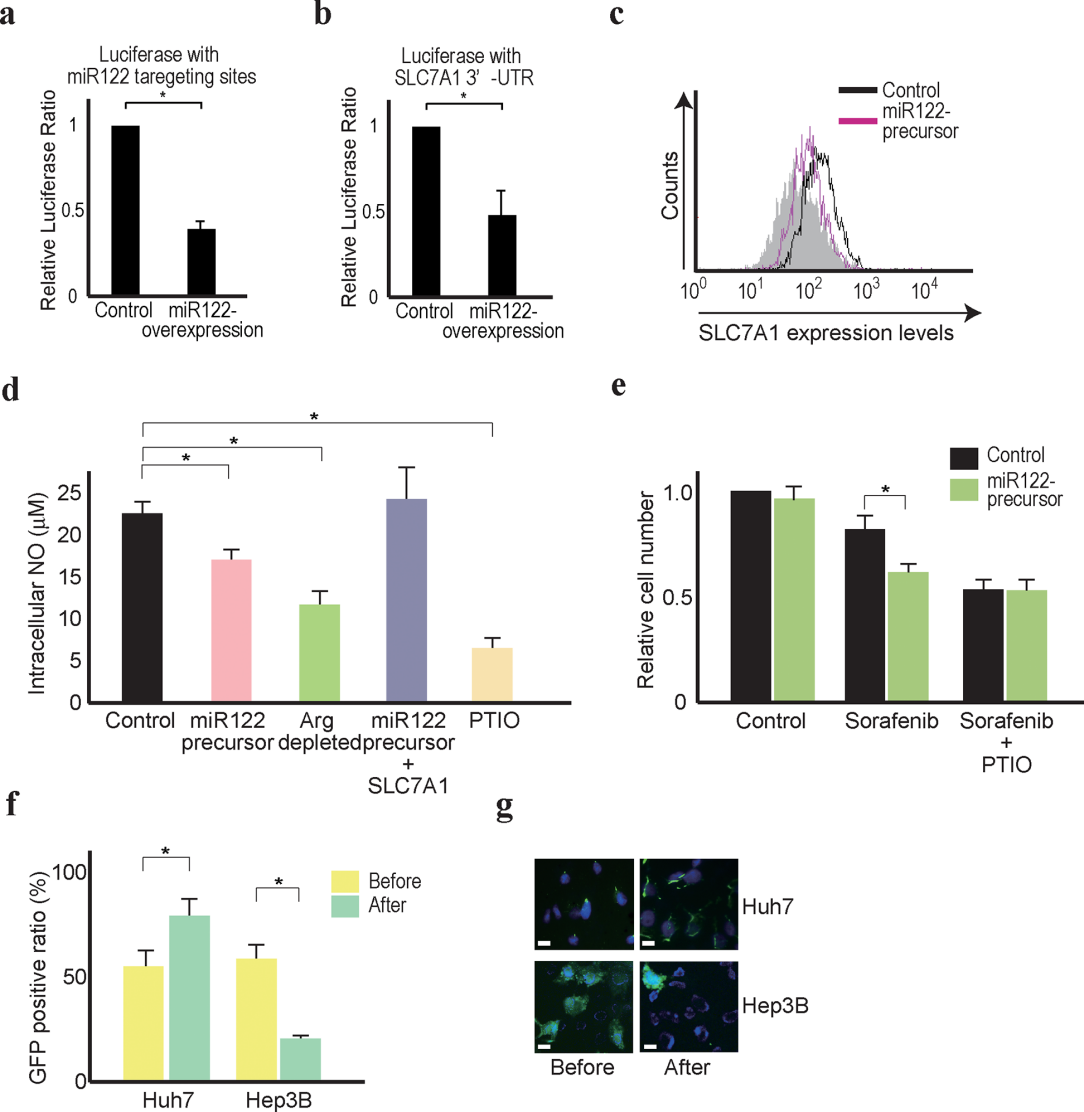
Forced miR122 expression sensitizes Hep3B cells to sorafenib treatment **(a, b)** The effects of miR122 on the reporter constructs carrying miR122 responsive elements (a) and the SLC7A1 3′UTR (b) in control and miR122 precursor-expressing Hep3B cells. Control cell values were set to 1. Data represent the means ± s.d. of three independent experiments. *, *p* < 0.05. **(c)** SLC7A1 expression was decreased in miR122 precursor-overexpressing Hep3B cells. Flow cytometry assessment of SLC7A1 protein expression was performed. Representative results from three independent experiments are shown. **(d)** Intracellular NO levels were decreased by miR122 precursor-expression in Hep3B cells. This effect was reversed by stable overexpression of HA-tagged SLC7A1. Control cells (control), miR122 precursor-overexpressing cells (miR122 precursor) and cells with stable overexpression of both the miR122 precursor and HA-tagged SLC7A1 (miR122 precursor + SLC7A1) were tested. Control cells under arginine-depleted conditions and treated with PTIO were included. *, *p* < 0.05 (*n* = 3). **(e)** The effects of sorafenib were inversely correlated with the expression levels of miR122. Relative cell numbers were counted after treatment for 48 h with 5 μM sorafenib with or without PTIO in miR122 precursor-expressing Hep3B cells. The number of the control cells with DMSO treatment was set to 1 *, *p* < 0.05 (*n* = 3). Control cells (control), miR122 precursor-expressing cells (miR122 precursor), cells cultured in arginine-depleted media (Arg depleted), and miR122 precursor-expressing cells with expression of SLC7A1 (miR122 precursor + SLC7A1) were tested. Cells treated with PTIO to remove NO were included as a control. **(f, g)** The effects of sorafenib were inversely correlated with the expression levels of miR122, as determined by flow cytometry (f) and fluorescence microscopy (g). miR122-silenced Huh7 cells and miR122 precursor-expressing Hep3B cells showed GFP co-expression. The percentage of GFP-expressing cells (~50% before sorafenib treatment) increased in the miR122-silenced Huh7 cells and decreased in the miR122 precursor-expressing Hep3B cells after 5 μM sorafenib treatment for 48 h, as determined by flow cytometry. *, *p* < 0.05 (*n* = 3) (f). Representative results of GFP-positive cells from three independent experiments are shown (g).

To determine the converse effects, the miR122 precursor was stably expressed in Hep3B cells, because these cells naturally expressed only minimal levels of miR122 [5]. Hep3B cells expressing the miR122 precursor showed enhanced miR122 function (Figure 4a) and suppressed luciferase expression from a reporter carrying the 3′ UTR of the SLC7A1 gene (Figure 4b), with lower expression of SLC7A1 protein compared with the control cells (Figure 4c). Furthermore, the cells showed lower intracellular NO levels, similar to those of cells grown under arginine-depleted culture conditions or treated with PTIO (Figure 4d). Because stable overexpression of SLC7A1 in the miR122 precursor-stably expressing Hep3B cells reversed the effects of intracellular NO contents, changes in intracellular NO levels appeared dependent on changes in the SLC7A1 expression levels (Figure 4d). It was noted that miR122 expression had a positive effect on the sorafenib-treated Hep3B cells, similar to the effects of removing NO with PTIO (Figure 4e). To exclude any possible influences of stable cell line specificity or differences in the culture conditions of the individual cell lines, Huh7 or Hep3B cells were transduced with miR122-silencing or miR122-overexpressing lentiviruses with GFP, but without selection. GFP-positive (miR122-silenced) Huh7 cells and GFP-negative (miR122-non-overexpressing) Hep3B cells were more concentrated after sorafenib treatment of mixtures of infected (GFP-expressing) and non-infected (non-GFP-expressing) cells cultured in bulk (Figure 4f and g), confirming that decreased miR122 function was related to sorafenib resistance. These results suggest that miR122-silenced HCC cells are more resistant to chemotherapeutic drugs, and that depletion of arginine in extracellular environments may be effective for the clinical management of an HCC subset with reduced miR122 expression levels.

### Identification of chemical compounds that enhanced miR122 expression levels

**Figure 5:**
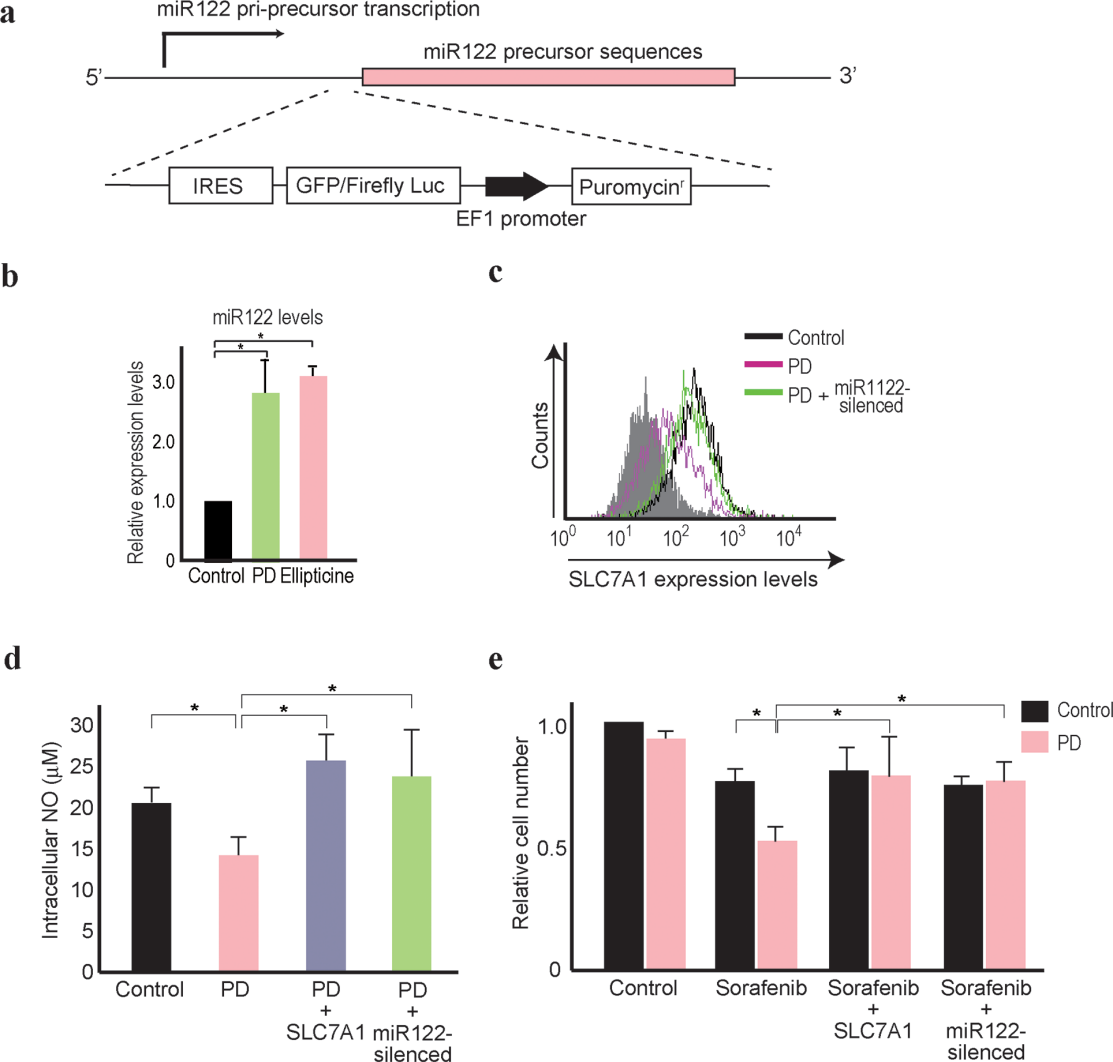
PD407824 increases miR122 expression and sensitizes Hep3B cells to sorafenib treatment **(a)** A schematic of the knock-in reporter at the miR122 pri-precursor locus. The arrow indicates the possible transcription start site. The gene-editing target site by Cas9 is located just before the miR122 precursor locus. Below, the knock-in construct is shown which consists of IRES, GFP fused with a firefly luciferase gene reporter, the EF1 promoter, and the puromycin resistant gene. **(b, c)** Mature miR122 levels in Hep3B cells after the treatment of PD407824 (PD) and Ellipticine for 24 h were measured by qRT-PCR. The levels from the control cells were set as 1.0. *, *p* < 0.05 (*n* = 3). nt, nucleotides. c, Flow cytometry results determining SLC7A1 protein expression levels. Representative results from three independent experiments are shown. SLC7A1 expression was decreased in Hep3B cells treated with PD407824 (PD), which was antagonized by expressing the antisense miR122 construct (PD + miR122-silenced). **(d)** Intracellular NO levels were decreased in Hep3B cells treated with PD407824. This effect was reversed by stable overexpression of HA-tagged SLC7A1. Control cells (Control), PD407824 treated cells (PD), and PD407824 treated cells with stable expression of HA-tagged SLC7A1 (PD + SLC7A1) or the antisense miR122 construct (PD + miR122-silenced) were tested. *, *p* < 0.05 (*n* = 3). **(e)** The effects of sorafenib were augmented by combination treatment with PD407824. Relative Hep3B cell numbers were counted after treatment with or without PD407824 (PD) for 48 h. Control cells (Control), sorafenib treated cells (Sorafenib), sorafenib-treated cells with stable expression of SLC7A1 (Sorafenib + SLC7A1) or stable expression of the antisense miR122 construct (Sorafenib + miR122-silenced) were tested. The number of control cells with DMSO (without sorafenib and PD407824) treatment was set to 1. *, *p* < 0.05 (*n* = 3).

The results obtained led us to hypothesize that if it were possible to enhance miR122 expression levels in HCC cells using chemical compounds, such compounds could augment the chemosensitivity of HCCs to sorafenib. To undertake a comprehensive screen of possible compounds, a knock-in reporter construct was created by inserting a firefly luciferase gene in the miR122 precursor genomic locus by genome-editing using the CRISPR-Cas9 system (Figure 5a and Supplementary Figure 4). These cells were screened with over 1,200 chemical compounds to identify molecules which potentially enhance miR122 pri-precursor transcription. Of several compounds that increased the reporter values (Supplementary Table 1), PD407824, a Wee1 kinase inhibitor, and Ellipticine, a DNA topoisomerase inhibitor, returned the highest values.

Using Hep3B cells, which naturally express low levels of miR122, the enhanced expression levels of miR122 after treatment with PD407824 or Ellipticine for 24 h were confirmed by quantitative RT-PCR (Figure 5b). Because the Wee1 kinase inhibitor was recently reported as a potential novel HCC therapeutic [37], our subsequent focus was on PD407824. SLC7A1 expression levels in Hep3B cells were suppressed by PD407824, and this was reversed by the stable expression of antisense miR122 construct (Figure 5c). Similarly, the NO content in the cell lysate was downregulated by PD407824, which was antagonized by the stable expression of SLC7A1 or antisense miR122 construct (Figure 5d). The number of Hep3B cells after combination treatment of sorafenib and PD407824 was significantly lower than that after sorafenib treatment alone (Figure 5e). While PD407824 itself may have anti-oncogenic effects on HCC cells [37], only marginal effects on Hep3B cell survival were observed in our study, probably due to the low concentration used (Figure 5e). The combinatorial effects of PD407824 and sorafenib were antagonized by the stable expression of SLC7A1 or the antisense miR122 construct (Figure 5e), suggesting that the synergistic effects of PD407824 with sorafenib on Hep3B cells were mediated by enhanced miR122 expression via PD407824. These results suggested that the combination of PD407824 and sorafenib may act favorably against HCCs through increasing miR122 expression levels.

## DISCUSSION

In this study, we demonstrate that miR122-silenced HCC cells and tissues have higher SLC7A1 expression, intracellular arginine, and NO contents, which may be linked to the sorafenib chemosensitivity, consistent with the previous reports that miR122 is linked with sorafenib sensitivity in HCC cells [38, 39].

A liver-specific miRNA, miR122, has been linked with pleiotropic physiological functions [14]. Notably, a remarkable decrease in the expression of this miRNA in HCCs is well documented and may play a crucial role in maintaining tumorigenic properties [4]. Recently, it is known that amino acid profiles are changed in cancers [40, 41]. Thus, to gain insights regarding the physiological effects on amino acid metabolism induced by miR122 silencing, changes in amino acid levels in miR122-silenced liver tissues were determined in this study. From the results of the initial screening, we focused on the increased intracellular arginine content with miR122 silencing, because SLC7A1, a well-known target of miR122 [27], is the transporter for arginine and the increased arginine levels were explained by changes in the expression levels of this gene.

SLC7A1, the first amino acid transporter for arginine to be cloned among CATs, has important metabolic functions, including in NO synthesis. Homozygous SLC7A1-knockout mice die on the first day after birth [42], suggesting an important physiological role for SLC7A1 by mediation of the basic arginine supply [43]. Interestingly, although SLC7A1 is expressed almost ubiquitously, its expression is absent in the adult normal liver [43], possibly because of high miR122 levels in the healthy liver. Therefore, it is possible that the decreased expression of miR122 in the liver in pathological states such as HCC may cause the abnormally increased expression of SLC7A1 and the subsequent metabolic changes due to amino acid imbalances.

L-arginine is the natural substrate of NO synthetase for the generation of NO [28, 36]. The availability of intracellular arginine is a rate-limiting factor in cellular NO production [44]. Despite the “arginine paradox”, in which extracellular and intracellular arginine concentrations may differ [36], CAT systems rely largely on concentration-dependent stimulation of transport by substrates on the opposite site of the membrane. Among CATs, CAT1 (SLC7A1) is the highest-affinity carrier of cationic amino acids, particularly L-arginine [43]. Thus, the increased expression of SLC7A1 may lead to increased intracellular arginine levels and subsequent NO production. Increased intracellular NO synthesis may function in a cell-autonomous manner in maintaining the cancer progenitor-cell properties, as was discovered recently in glioma [30]. In fact, human breast cancer cell lines were reported to promote cell survival through arginine-uptake via CAT1 expression [45]. Other reports suggest that endogenously produced NO is generally cytoprotective [46, 47]. Therefore, depletion of NO by blocking the related pathway, for example by NO removal, NOS inhibition, modulation of SLC7A1 expression, or arginine depletion, may have therapeutic effects on HCCs.

Another important finding of this study was the identification of those compounds that enhanced transcription of the miR122 precursor. Although the molecular mechanisms of how those compounds enhanced the miR122 precursor transcription remain unclear, combination therapy with sorafenib may provide a promising approach to the treatment of resistance in HCC by enhancing miR122 expression and reducing SLC7A1 levels. In addition, the compounds identified also possess anti-cancer properties at appropriate doses, irrespective of the effects of increased miR122 expression levels, which may also be beneficial for the treatment of HCCs in combination with sorafenib. Because these experiments are performed in *in vitro* studies using two kinds of HCC cell lines in this study, it is important to test other cell lines and also *in vivo* experiments in the future for the future clinical application of these findings.

Arginine deprivation using pegylated arginine deiminase is currently undergoing a phase 3 trial for HCC and melanoma [48–51]. This clinical trial is based on the fact that tumor cells cannot grow in media depleted of arginine [52]. Our results may provide a basic rationale for HCC treatments targeting arginine, especially in the presence of reduced miR122 expression. Given the series of failures of recent phase 3 trials of various molecular-targeted agents in HCC due to the lack of predictive biomarkers of a response, our results show promise for the development of personalized/stratified HCC therapy.

In summary, we have shown that decreased miR122 expression in HCC leads to increased intracellular arginine and NO levels through elevated SLC7A1 expression, which may be involved in chemoresistance. Our results suggest that deprivation of arginine or combination of sorafenib and miR122 expression-enhancing drugs may be useful in the management of a subset of HCCs with reduced miR122 expression levels.

## METHODS

### Cell culture

The human hepatocellular carcinoma cell lines Huh7 and Hep3B cells, and human embryonic kidney cell line 293T cells, were obtained from the American Type Culture Collection (ATCC, Rockville, MD, USA). Cells were maintained in Dulbecco's modified Eagle's medium (DMEM) supplemented with 10% fetal bovine serum, unless otherwise specified.

### Mouse experiments

MiR122 functionally silenced transgenic C57BL/6 mice which expressed antisense miR122 oligonucleotides under the control of the H1 promoter were described in detail in our previous study [5]. Mouse experimental protocols were approved by the Ethics Committee for Animal Experimentation at the University of Tokyo (#13-P-54), and experiments were conducted in accordance with the guidelines for the care and use of laboratory animals of the University of Tokyo.

### Reagents and arginine-depleted media

2-(4-Carboxyphenyl)-4,4,5,5-tetramethylimidazoline-1-oxyl-3-oxide [carboxy-PTIO, a stable and potent NO scavenger] was purchased from Dojindo Laboratories (Kumamoto, Japan). Arginine-depleted DMEM was specially formulated and provided by Ajinomoto Pharmaceuticals (Tokyo, Japan). Fetal bovine serum was not added when using this medium. Sorafenib was purchased from Toronto Research Chemicals (North York, ON, Canada), and dissolved in dimethylsulfoxide (DMSO) as a 5 mM stock. An equal volume of DMSO was used as the negative control. PD407824, a Wee1 and Chk1 inhibitor, and Ellipticine, a DNA topoisomerase inhibitor, were purchased from TOCRIS Bioscience (Bristol, UK) and used at 1 μM.

### Antibodies

The antibodies used included anti-CAT1 (ab37588), purchased from Abcam (Cambridge, UK), and anti-EpCAM (#2929), anti-iNOS (#2977), anti-eNOS (#9572), and anti-nNOS (#4234), all purchased from Cell Signaling Technology (Danvers, MA, USA). Anti-CD13 (#301701) was purchased from BioLegend (San Diego, CA, USA). Anti-β-actin (#A5441) was purchased from Sigma (St. Louis, MO, USA). Isotype IgG antibodies were purchased from R&D Systems (Minneapolis, MN, USA).

### Comprehensive amino acid analyses

Liver tissues from two control C57BL/6 mice and from two miR122-silenced transgenic mice were subjected to comprehensive analyses of amino acid levels, as described previously [53]. Briefly, pre-weighed, snap-frozen tissues were homogenized using a cell disrupter (MS-100R; TOMY, Tokyo, Japan) at 2°C after adding 500 μL of methanol containing internal standards [methionine sulfone, 2-(N-morpholino)-ethanesulfonic acid (MES), and D-camphol-10-sulfonic acid (CSA), 20 μM each]. The homogenate was then mixed with 20 μL of Milli-Q water and 500 μL of chloroform and centrifuged at 4,600 g for 15 min at 4°C. Subsequently, 300 μL of the aqueous solution were centrifugally filtered through a 5-kDa cut-off filter (Millipore, Bedford, MA, USA) to remove any proteins. The filtrate was centrifugally concentrated and dissolved in 50 μL of Milli-Q water containing reference compounds (3-aminopyrrolidine and trimesate, 200 μM each) immediately prior to capillary electrophoresis time-of-flight mass spectrometry (CE-TOFMS) analysis.

Cationic amino acids were separated in a fused-silica capillary (i.d. 50 μm, total length 100 cm) filled with 1 M formic acid as the reference electrolyte [54]. Sheath liquid comprising methanol/water (50%/50%, v/v) and 0.5 μM reserpine was administered at 10 μL/min. The ESI-TOFMS was operated in positive ion mode. Separation of anionic amino acids was performed in a cationic polymer-coated SMILE (+) capillary (Nacalai Tesque, Kyoto, Japan) filled with 50 mM ammonium acetate solution (pH 8.5) [55]. The sample solution was injected at 50 mbar for 30 s, and a negative voltage of −30 kV was applied. Ammonium acetate (5 mM) in methanol/water (50%/50%, v/v) containing 1 μM reserpine was administered as the sheath liquid at 10 μL/min. The ESI-TOFMS was operated in negative ion mode. Other conditions were as described previously [53]. For the quantitation of the amino acids in cultured cells, cells were washed with PBS and lysed with an 8:2 methanol: water solution. Chloroform was then added and the mixture was centrifuged. The resulting supernatant was stored at −80°C until analysis. Intracellular amino acids (arginine and phenylalanine) in the cell lysates were quantified as described previously [56].

### Western blotting, transfection, and dual luciferase assays

Western blotting, transfection and dual luciferase assays were performed as described previously [5]. Briefly, protein lysates were prepared from cells for Western blotting. Proteins were separated by SDS-polyacrylamide gel electrophoresis and transferred to polyvinylidene difluoride membranes. After blocking with 5% dry milk to decrease non-specific binding, the membranes were probed with the appropriate primary antibodies. Horseradish peroxidase-conjugated secondary antibodies were used to detect primary antibodies. Bound antibodies were detected using ECL Plus Western Blotting Detection Reagents (GE Healthcare Life Sciences, Pittsburgh, PA). All plasmid transfections were performed using FuGENE 6 Transfection Reagent (Boehringer Mannheim, Mannheim, Germany) according to the manufacturer's instructions. pGL4-TK, a control plasmid containing *Renilla reniformis* (sea pansy) luciferase under the control of the herpes simplex virus thymidine kinase promoter (Toyo Ink, Tokyo, Japan) was used to determine the transfection efficiency. Relative luciferase values were calculated by normalizing firefly luciferase activity values to sea pansy luciferase activity values to account for changes in transfection efficiency. Luciferase activity was measured using the Dual-Luciferase Reporter Assay System (Promega, Madison, WI, USA) with a luminometer (Lumat LB9507; EG&G Berthold, Bad Wildbad, Germany).

### Plasmids, virus production, and transduction

The firefly luciferase-based reporter carrying a miR122-responsive element in its 3′ untranslated region (UTR), used to examine miR122 function, and the internal control Renilla luciferase-based plasmids have been described previously [5]. A Renilla luciferase-based reporter constructed with the SLC7A1 3′UTR, which contains three miR122 responsive elements, was kindly provided by Prof. Filipowicz [27]. When using this construct, the CMV promoter-driven firefly luciferase construct (Promega) was used as an internal control. The miR122 precursor in the eGFP-expressing plasmid (pCDH-miR122 with eGFP) and the H1 promoter-driven antisense miR122 stem-loop-stem RNA-expressing plasmid (pmiRZIP122 with eGFP) were purchased from System Biosciences (Mountain View, CA, USA). The miR122 precursor-expressing plasmid with the puromycin resistance gene (pCDH-miR122 with puro), which was constructed by replacing the eGFP gene with the puromycin resistance gene via the *Fse*I site, was constructed as described previously [5]. pmiRZIP122 without the eGFP gene (pmiRZIP122 with puro) for miR122 silencing was constructed by excision of the eGFP-coding sequences via the *Xba* I and *Pst* I sites, followed by ligating the cut ends annealed with oligonucleotides (5′-CTA GAC GCC ACC ATG CTG CA-3′ and 5′-GCA TGG CGT-3′) to maintain the expression of the downstream puromycin resistance gene. An HA-tagged SLC7A1-expressing a lentiviral construct (pCDH-HA-SLC7A1) was made by an infusion technique (Clontech, Mountain View, CA) by inserting a PCR-amplified HA-tagged SLC7A1 cDNA using a Halo-tagged SLC7A1-expressing vector purchased from Promega (FHC24970 clone) as the template. The primers used were 5′-ATG TTC CAG ATT ACG CTA TGG GGT GCA AAG TGG TGC TC-3′ and 5′-TTA AAC CTT GCA CTG GTC CAA GT-3′. SLC7A1 knockdown construct shSLC7A1-expressing lentiviral particles were purchased from Santa Cruz Biotechnology (Dallas, TX). The pCDH control vector (System Biosciences) was used as a negative control. Lentiviral particles were produced using a pPACKH1 lentivector packaging plasmid mix, according to the manufacturer's recommendations (System Biosciences). Cells were transduced with lentiviruses using polybrene (EMD Millipore, Billerica, MA, USA) and then selected with puromycin, unless otherwise specified.

### Compound screening

To screen those chemical compounds that had the potential to increase miR122 precursor transcription, knock-in reporter cells were prepared using the CRISPR/Cas9 gene editing system. The targeting site by the guide RNA with the protospacer adjacent motif (PAM) sequences at the 11 bases upstream of miR122 precursor lesions was determined using the CRISPRdirect database (http://crispr.dbcls.jp/). The target sequences AGG TGA AGT TAA CAC CTT CGT GG were cloned into the Cas9 SmartNulcease tagged vector (System Biosciences), which expressed the guide RNA under the H1 promoter and humanized Cas9 under the CAG promoter from a single vector. For the donor construct, the firefly luciferase gene was amplified by PCR using the pGL4.50 vector (Promega) as a template and inserted and fused to the original eGFP gene at the Bsp1407I site of the HR180PA-1 targeting vector (System Biosciences) by the Infusion method, to construct a luciferase-based reporter donor vector. Approximately 1,600-base-pair sequences around the Cas9 targeting site were inserted before and after the reporter construct as 5′ and 3′ homology arms, respectively. Because this construct has a promoter-less IRES element upstream of the eGFP and firefly luciferase gene, eGFP and luciferase mRNA were transcribed by the host gene promoter at the knock-in locus, and the proteins were translated from the IRES. Thus, the reporter expression levels reflected the transcription levels of the miR122 pri-precursor lesion. Since this donor vector also contained a puromycin-resistance gene driven by an independent EF1 promoter between the 5′ and 3′ homology arms, the cells containing the successfully edited gene could be selected by puromycin. Because 293T cells also express miR122 [57] and these cells were efficient at gene-editing, the above Cas9 expressing construct and the donor vector were transfected into 293T cells and selected with 2 μg/ml puromycin; subsequently 12 colonies were picked up. Genomic DNA was extracted from the colonies and the correct knock-in was confirmed by the 5′ and 3′ junction PCR method. The genomes of the cells from all colonies picked up were edited successfully.

A chemical compound library (LOPAC1280) containing 1,280 compounds was purchased from Sigma-Aldrich (St. Louis, MO). Reporter knocked-in cells were seeded in 96-well plates and 1 μM of compounds in DMSO were added for 24 h. Luciferase values were measured by a GLOMAX 96-well microplate luminometer (Promega) and relative values were calculated by adjusting the average values from the DMSO-treated control cells in eight wells in each plate as 1.0.

### Flow cytometry

Cells were hybridized with the primary antibody or the isotype control IgG in 5% BSA/1% sodium azide/PBS for 1 h at 4°C. After washing, the cells were incubated with a secondary antibody conjugated with Alexa 488 (1:1000; Molecular Probes, Eugene, OR, USA) for 30 min. Flow cytometry was performed and the data analyzed using Guava EasyCyte Plus (GE Healthcare, Little Chalfont, UK), as described previously [58].

### Cell counting and NO quantitation

Relative cell proliferation was assessed using the Cell Counting Kit-8 (Dojindo Laboratories) as described previously [5]. For drug testing, relative cell numbers were counted after treatment for 48 h either with sorafenib (5 μM), with PD407824 (1 μM), with Ellipticine (1 μM) or with combinations thereof. The cell numbers were calculated by comparison to the control cells treated with DMSO. NO quantitation in the cell lysate from cells treated with arginine-depletion, PTIO treatment, or other compound treatments for 24 h was performed using the QuantiChrom Nitric Oxide Assay Kit (BioAssay Systems, Hayward, CA, USA) according to the manufacturer's instructions.

### Fluorescence microscopy

To observe GFP expression, cells were transferred to a Lab-Tek chamber and mounted with Vectashield containing DAPI (Vector Laboratories, Burlingame, CA, USA). Cells were observed before and after sorafenib treatment using a fluoromicroscope (Olympus, Tokyo, Japan).

### miRNA quantitation by qRT-PCR and Northern blotting

Total RNA was extracted using TRIzol Reagent (Invitrogen, Carlsbad, CA) according to the manufacturer's instructions. Northern blotting of miRNAs was performed as described previously [59]. Briefly, 10 μg of RNA were resolved in a denaturing 15% polyacrylamide gel containing 7 M urea in 1× TBE and then transferred to a Hybond N+ membrane (GE Healthcare) in 0.25 × TBE. Membranes were UV-crosslinked and prehybridized in hybridization buffer. Hybridization was performed overnight at 42°C in ULTRAhyb-Oligo Buffer (Ambion) containing a biotinylated probe specific for miR122 (caa aca cca ttg tca cac tcc a), which had previously been heated to 95°C for 2 min. Membranes were washed at 42°C in 2 × SSC containing 0.1% SDS and the bound probe was visualized using a BrightStar BioDetect Kit (Ambion). Blots were stripped by boiling in a solution containing 0.1% SDS and 5 mM EDTA for 10 min prior to rehybridization with a U6 probe (cac gaa ttt gcg tgt cat cct t). To measure the amount of miR122 in the cells, a miRCURY LNA microRNA qPCR system (Exiqon, Vedbaek, Denmark) was used according to the manufacturer's instructions. The levels of U6 snRNA were used for normalization of cellular miRNA levels.

### Analysis of clinical HCC cohorts

To determine the clinical relevance of miR122 deregulation on SLC7A1 expression, previously generated genome-wide mRNA and miRNA profiles of a hepatitis B-related HCC cohort (*n* = 192) [20, 21] and a hepatitis C-related HCC cohort (*n* = 89) [22, 23] (NCBI Gene Expression Omnibus; accession numbers GSE6857, GSE14520, GSE20594, and GSE9843) were analyzed. Pre-normalized data in the respective publications were summarized by calculating the median of multiple probes corresponding to the SLC7A1 gene.

### Statistical analysis

Statistically significant differences between groups were identified using Welch's *t*-test. *P* values less than 0.05 for *in vitro* experiments were considered to indicate statistical significance. The correlation between SLC7A1 and miR122 expression levels was assessed by Spearman correlation tests.

## SUPPLEMENTARY FIGURES AND TABLE




