# Supplementary material for: Decreased miR122 in hepatocellular carcinoma leads to chemoresistance with increased arginine
Source: Oncotarget. 2015 Mar 21;6(10):8339–52. doi: 10.18632/oncotarget.3234 (PMC4480756; doi:10.18632/oncotarget.3234)
Supplement: Supplementary file 1 [file oncotarget-06-8339-s001.pdf]

SUPPLEMENTARY FIGURES AND TABLE

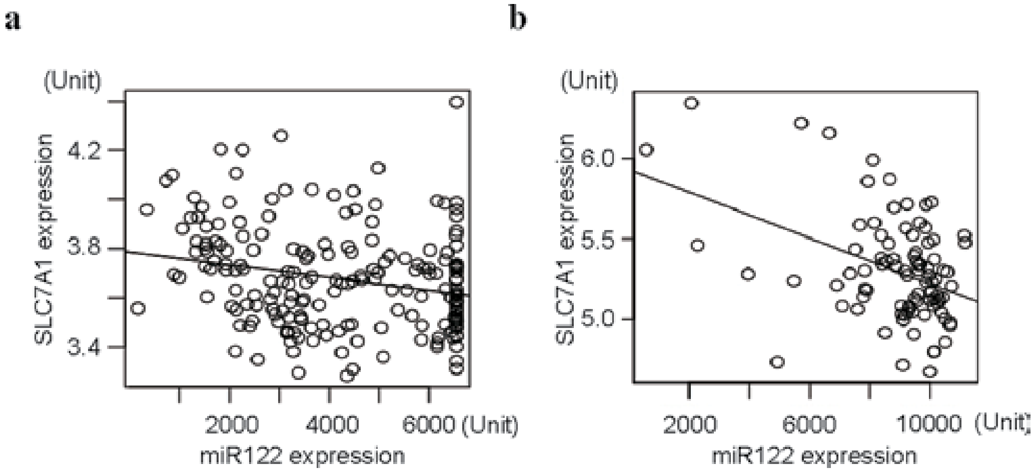

Supplementary Figure 1: Correlation between miR122 and SLC7A1 expression levels in hepatitis-related HCC cohorts. Negative correlation between miR122 and SLC7A1 expression levels in **(a)** a hepatitis B-related HCC cohort (n = 192) (Spearman's rho = −0.239, $p < 0.001$) and **(b)** a hepatitis C-related HCC cohort (n = 89) (Spearman's rho = −0.289, $p < 0.001$).

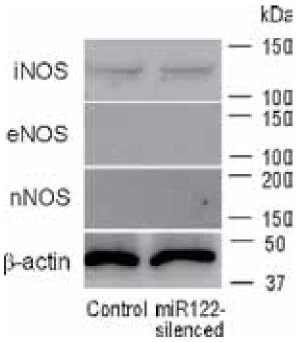

Supplementary Figure 2: Intracellular iNOS levels were unchanged in miR122-silenced Huh7 cells. The expression levels of three NO synthetases were determined by Western blotting. eNOS and nNOS were not detected in Huh7 cells. iNOS levels were not changed significantly by miR122 silencing.

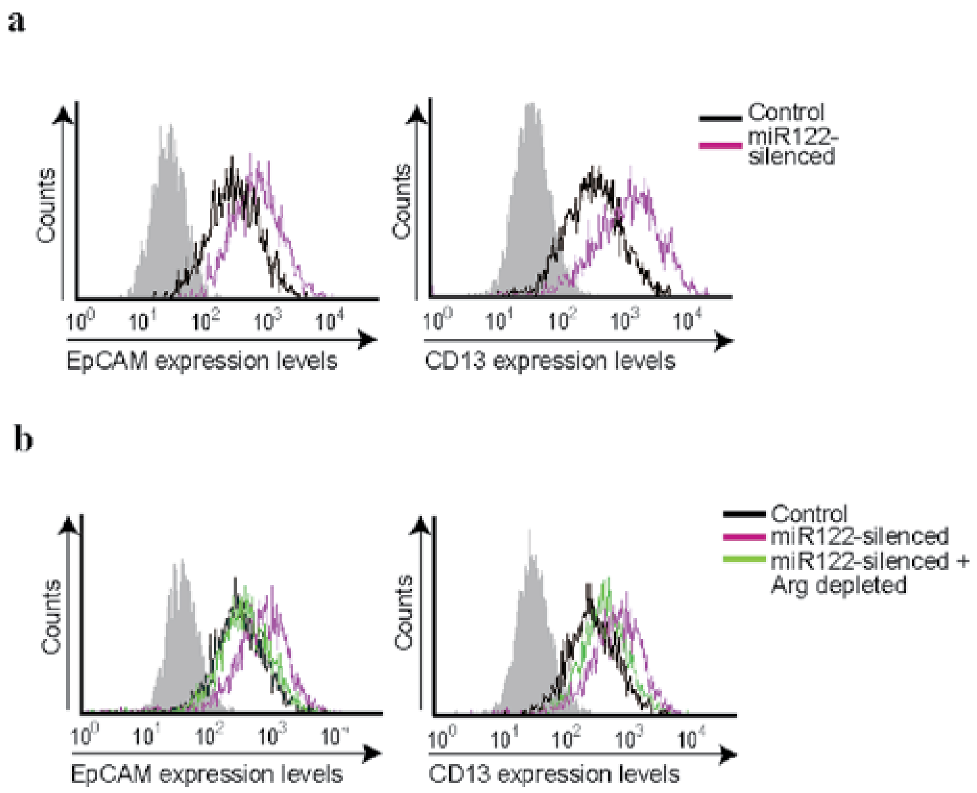

Supplementary Figure 3: EpCAM and CD13 expression levels were increased in miR122-silecend cells but decreased in cells cultured in arginine-depleted media. Flow cytometry assessment of protein expression levels in control cells (black line), miR122-silenced cells (pink line), and miR122-silenced cells under arginine-depleted conditions (green line). Gray-shaded histograms represent the isotype IgG background. Representative results from three independent experiments are shown. **(a)** EpCAM and CD13 expression levels were increased in miR122-silenced Huh7 cells. **(b)** Increased EpCAM and CD13 expression levels were antagonized in miR122-silenced Huh7 cells under arginine-depleted conditions.

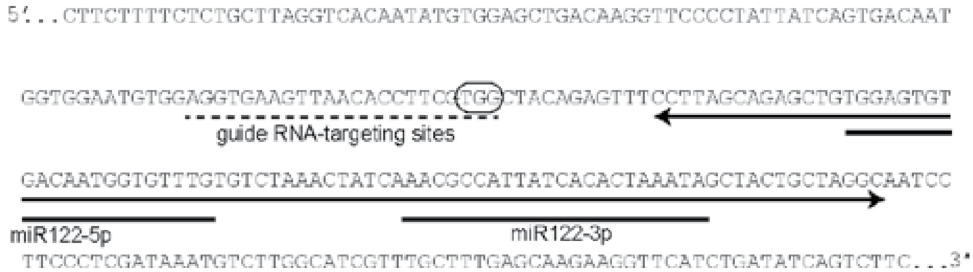

Supplementary Figure 4: miR122 genomic locus and Cas9 targeting site. Genomic sequences around the miR122 precursor locus. Line with arrowheads indicates miR122 precursor genomic sequences. Bold lines indicate mature miR122-5p and miR122-3p. The dashed line indicates the guide RNA-targeting sequences for Cas9-mediated cleavage. "TGG" indicates the PAM sequences. Target sequences were potentially located in the miR122 pri-precursor sequences, which were transcribed from the genome for the expression of miR122.

Supplementary Table 1: Summary of the results of the compound library screening for increasing miR122 transcription.
